# hSSB2 (NABP1) is required for the recruitment of RPA during the cellular response to DNA UV damage

**DOI:** 10.1038/s41598-021-99355-0

**Published:** 2021-10-12

**Authors:** Didier Boucher, Ruvini Kariawasam, Joshua Burgess, Adrian Gimenez, Tristan E. Ocampo, Blake Ferguson, Ali Naqi, Graeme J. Walker, Emma Bolderson, Roland Gamsjaeger, Kenneth J. O’Byrne, Liza Cubeddu, Kum Kum Khanna, Derek J. Richard

**Affiliations:** 1 Cancer & Ageing Research Program, Centre for Genomics and Personalised Health at the Translational Research Institute (TRI), Queensland University of Technology (QUT), Brisbane, Australia; 2 School of Science and Health, Western Sydney University, Penrith, NSW 2751 Australia; 3 School of Life and Environmental Sciences, University of Sydney, Sydney, NSW 2006 Australia; 4 Drug Discovery Group, QIMR Berghofer Medical Research Institute, Herston, QLD 4006 Australia; 5 Department of Chemistry, Pennsylvania State University, University Park, USA; 6 Diamantina Institute, University of Queensland, Woolloongabba, QLD 4102 Australia; 7 Princess Alexandra Hospital, Woolloongabba, QLD 4102 Australia; 8 Signal Transduction Group, QIMR Berghofer Medical Research Institute, Herston, QLD 4006 Australia

**Keywords:** Stress signalling, Genomic instability, Cancer prevention

## Abstract

Maintenance of genomic stability is critical to prevent diseases such as cancer. As such, eukaryotic cells have multiple pathways to efficiently detect, signal and repair DNA damage. One common form of exogenous DNA damage comes from ultraviolet B (UVB) radiation. UVB generates cyclobutane pyrimidine dimers (CPD) that must be rapidly detected and repaired to maintain the genetic code. The nucleotide excision repair (NER) pathway is the main repair system for this type of DNA damage. Here, we determined the role of the human Single-Stranded DNA Binding protein 2, hSSB2, in the response to UVB exposure. We demonstrate that hSSB2 levels increase in vitro and in vivo after UVB irradiation and that hSSB2 rapidly binds to chromatin. Depletion of hSSB2 results in significantly decreased Replication Protein A (RPA32) phosphorylation and impaired RPA32 localisation to the site of UV-induced DNA damage. Delayed recruitment of NER protein Xeroderma Pigmentosum group C (XPC) was also observed, leading to increased cellular sensitivity to UVB. Finally, hSSB2 was shown to have affinity for single-strand DNA containing a single CPD and for duplex DNA with a two-base mismatch mimicking a CPD moiety. Altogether our data demonstrate that hSSB2 is involved in the cellular response to UV exposure.

## Introduction

Genetic stability is constantly challenged by endogenous and exogenous sources of DNA damage. Ultraviolet (UV) light is the primary cause of exogenous damage in our skin cells. There is now undeniable evidence that UV exposure is linked to skin cancer and photo-ageing^1,2^. With UVC (100–280 nm) filtered by the ozone layer, the UV spectrum on Earth at sea level is composed of UVB (280–315 nm) and UVA (315–400 nm). UVB radiation is absorbed directly by DNA, generating highly mutagenic cyclobutane pyrimidine dimers (CPD) and 6–4 photoproducts^3,4^. UVA, however, mainly generates oxidative damage to DNA^5^.

The main pathway to repair UVB and UVC induced DNA damage is the nucleotide excision repair (NER) pathway^6^. UV damage within the genome can be repaired by two distinct but over lapping sub-pathways of NER. The global genome repair pathway (GG-NER^7^) is initiated by the binding of the Xeroderma Pigmentosum group C (XPC)-HR23B complex. This complex together with the DDB1-DDB2 complex, senses the oscillation defect of the DNA sequence containing the UV damage^8^. This is followed by the recruitment of the TFIIH complex, XPA and the heterotrimer RPA (Replication Protein A). This complex allows the stabilisation of the nucleotide containing the damage and preparation for its removal by the recruitment of the endonuclease XPG, specifically incising the 3′ end after incision of the 5′ end by the endonuclease XPF with ERCC1. Once the oligonucleotide is removed (24–32 nt), a new DNA sequence is synthesised by the DNA replication machinery. The ligation of the new DNA fragment is performed by DNA ligase I. For transcription coupled repair (TC-NER^9^), the damage is recognized at a stalled RNA polymerase II site, promoting the recruitment of the CSA and CSB proteins. Recruitment of TFIIH, XPA and RPA follows, as described for the GGR. TCR is restricted to transcribed regions of the genome.

The importance of the NER pathway for the repair of UV induced DNA damage is observed in the Xeroderma Pigmentosum syndrome where patients show severe sensitivity to UV with rapid development of skin cancer^10,11^.

In parallel to the onset of the repair process, UV damage also induces a signal transduction response, activating the ATR kinase (Ataxia Telangiectasia Mutated Rad-3 Related) checkpoint pathway^12^. Upon recruitment through its interacting protein (ATRIP) and the proteins RPA and TopBP1^13,14^, ATR activates, with its effecter Chk1, a wide range of proteins involved in DNA repair, cell cycle regulation and apoptosis^15^, including the RPA heterotrimer^16,17^.

A common feature of all DNA repair processes is the transient generation of single-stranded DNA (ssDNA). This ssDNA must be protected from nuclease and free radical attack to avoid loss of genetic information. This is the role of the “single-stranded DNA binding protein” family members. These proteins are critical for cellular survival and are ubiquitous to all life. They are characterised by a structurally conserved Oligonucleotide/Oligosaccharide-Binding (OB) fold. The family can be further subdivided into the “simple SSBs” and the “RPA” subfamilies^18–23^. SSBs from the three domains of life share little sequence similarity and diverse subunit organisation, but a common evolutionary feature of this SSB protein family is the capability of very different protein sequences to adopt a conserved OB fold (five-stranded beta-sheet coiled to form a closed beta-barrel) to bind ssDNA with high affinity^24,25^. The roles of SSBs in DNA repair has been described in all three domains of life^22,26–30^. In humans, RPA has been well described as a critical ssDNA binding protein required for DNA repair^16^. While it was originally believed RPA was the only SSB family member functioning within the eukaryotic nucleus we have identified two members of the simple SSB subfamily encoded within the human genome, which have been named hSSB1 and hSSB2 (also known as OBFC2B, NABP2 (hSSB1) and OBFC2A, NABP1 (hSSB2)). The role of hSSB1 has been characterized for the repair of DNA double-strand breaks^31–34^, DNA replication forks^35^, and more recently DNA oxidative damage^36,37^, and also for the protection of telomeres^38^ and RNA processing^39^. In support of a role in ATR-mediated pathways, Kar et al.^40^ also observed that hSSB1-hSSB2 can trigger the ATR signalling pathway in the absence of RPA. hSSB1 has also been shown being a substrate of ATR kinase^41^. However, hSSB2 lacks ATR consensus phosphorylation site.

hSSB1 and hSSB2 differ by their C-terminal tail^42^ and can bind the Integrator complex Subunit 3 (IntS3) to form a complex^32–34^. Loss of IntS3 results in a loss of the hSSB1 transcript and protein^39^ suggesting this complex functions predominantly in transcriptional regulation.

The absence of SSB1 in mice leads to growth retardation, and increased genome instability^38,43,44^. However, little is known about hSSB2. hSSB2 has been shown to be involved in DNA double-strand breaks repair^32,33,45^, notably being required for the recruitment of Rad51^46^. No defect has been observed in SSB2 knock-out mice^47^, but a double knockout SSB1 and SSB2 mouse has a more severe phenotype than SSB1 or SSB2 alone, indicating the potential for each to compensate the loss of the other^48^. More recently, we have shown that hSSB2 is able to recognize and bind to pyrimidine dimers^49^.

In this current study we have sought to determine if hSSB2 has a role to play in other DNA repair pathways and in particular the cellular response to UVB radiation. Here we show that hSSB2 is rapidly stabilized after UVB irradiation (in vitro as well as in vivo), transiently binds to the chromatin and is required for the recruitment of RPA, indicating that hSSB2 is involved in the early response to UV exposure.

## Results

### hSSB2 protein levels increase following exposure to UVB, in vitro and in vivo

While hSSB1 has been demonstrated to function in the repair of double strand DNA breaks, stalled DNA replication forks and the repair of oxidative DNA damage^31,34–36,50^, the role of hSSB2 has not been as thoroughly characterised. We and others have previously shown that hSSB2 plays a role in the repair of DNA double-strand breaks^33,34^, however it is not yet clear if it plays additional roles in other repair pathways. We thus wanted to determine if hSSB2 responded to other forms of DNA damage.

**Figure 1:**
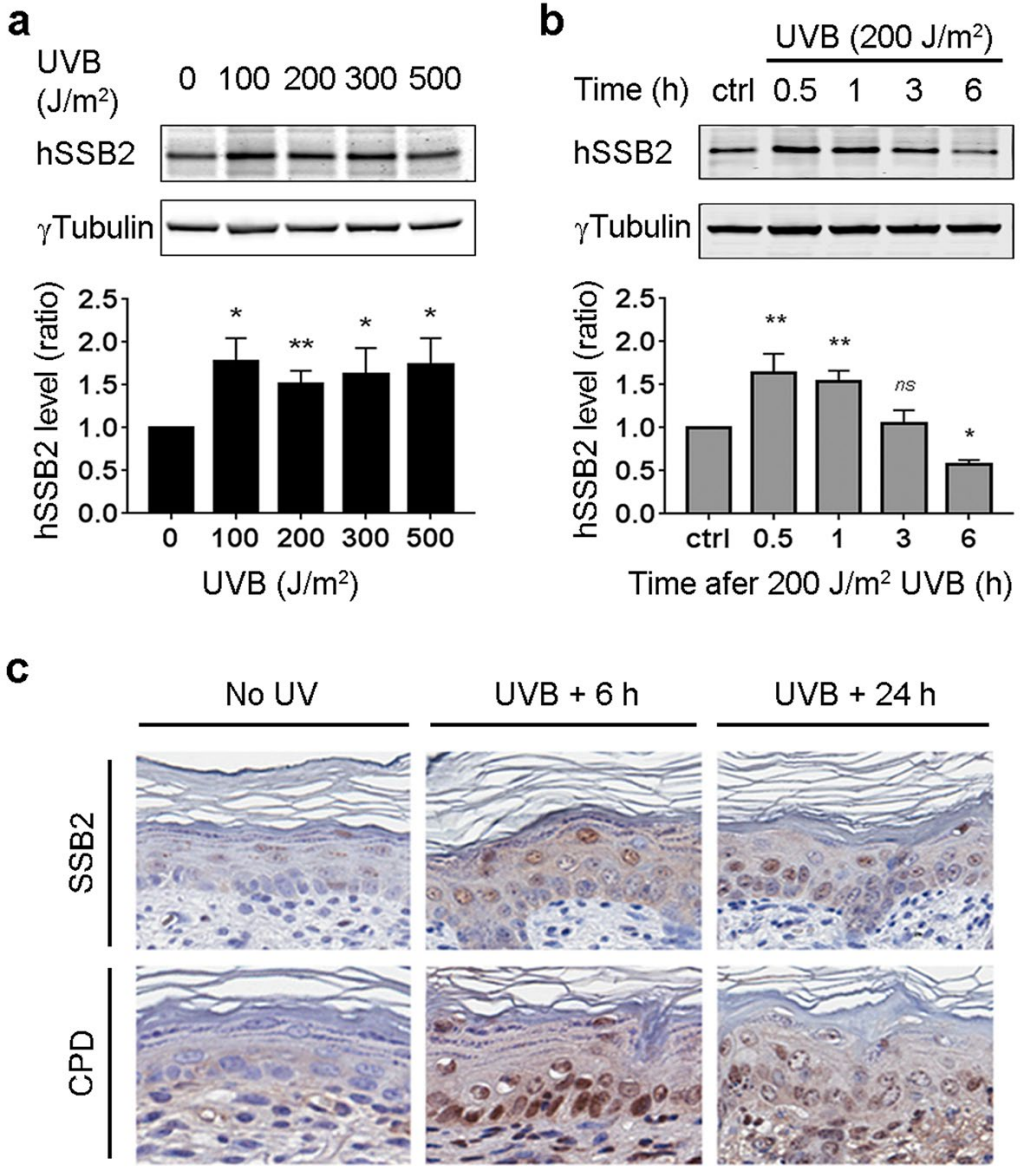
hSSB2 level increases after UVB exposure. (**a**,**b**) Representative western blot for hSSB2 and $\upgamma$ Tubulin for HeLa cells protein extracts 1 h after increasing doses of UVB (**a**) and at increasing repair time (h) after 200 J/m^2^ UVB (**b**). Average hSSB2 levels (± sem) over 3 independent experiments have been reported in the graphs below the blots (*ns*: non-significant; *P-value < 0.05; **P-value < 0.01; ***P-value < 0.001 in a One-Way ANOVA). (**c**) Immuno-histo chemistry staining (brown staining) for SSB2 (top panels) and DNA UV damage (CPD, bottom panels) of skin sections from 3 days old C57BL/6 mice not exposed to UV (left panels), 6 h (middle panels) and 24 h (right panels) post UVB exposure.

To determine if hSSB2 was involved in the repair of UVB generated DNA lesions, we first exposed HeLa cells to UVB irradiation. Following DNA damage, some repair proteins can become stabilised. For example hSSB1 is rapidly stabilised following the generation of double strand DNA breaks^31,33,34^. Using western blotting we observed a 1.5-fold increase in hSSB2 protein 1 h after various doses of UVB irradiation (Fig. 1a).

To explore this response further, HeLa cells were exposed to 200 J/m^2^ of UVB irradiation and the response of hSSB2 measured over time. Stabilization of hSSB2 occurred within 30 min, however, the levels returned to normal at 3–6 h post UVB exposure (Fig. 1b). To determine if the same response could be observed in an in vivo mouse model we used skin sections from wild-type C57BL/6 mice (3 days old^51^) exposed to UVB and allowed to recover for 6–96 h. Using immuno-histochemistry (IHC) we stained the sections for SSB2 (mouse hSSB2) (Fig. 1c, top panels and Supplementary Figure 1) and UVB induced DNA damage pyrimidine dimers (CPD, Fig. 1c—bottom panels) as a control of irradiation. We used *Ssb2*−/− mouse skin^47^ to validate the specificity of SSB2 labelling (data not shown). We observed that, as for HeLa cells in vitro, SSB2 level increased in the dermis of skin exposed to UVB. This indicated for the first time that hSSB2 may be involved in the response to UV induced DNA damage.

### hSSB2 rapidly binds to chromatin after UV exposure

**Figure 2:**
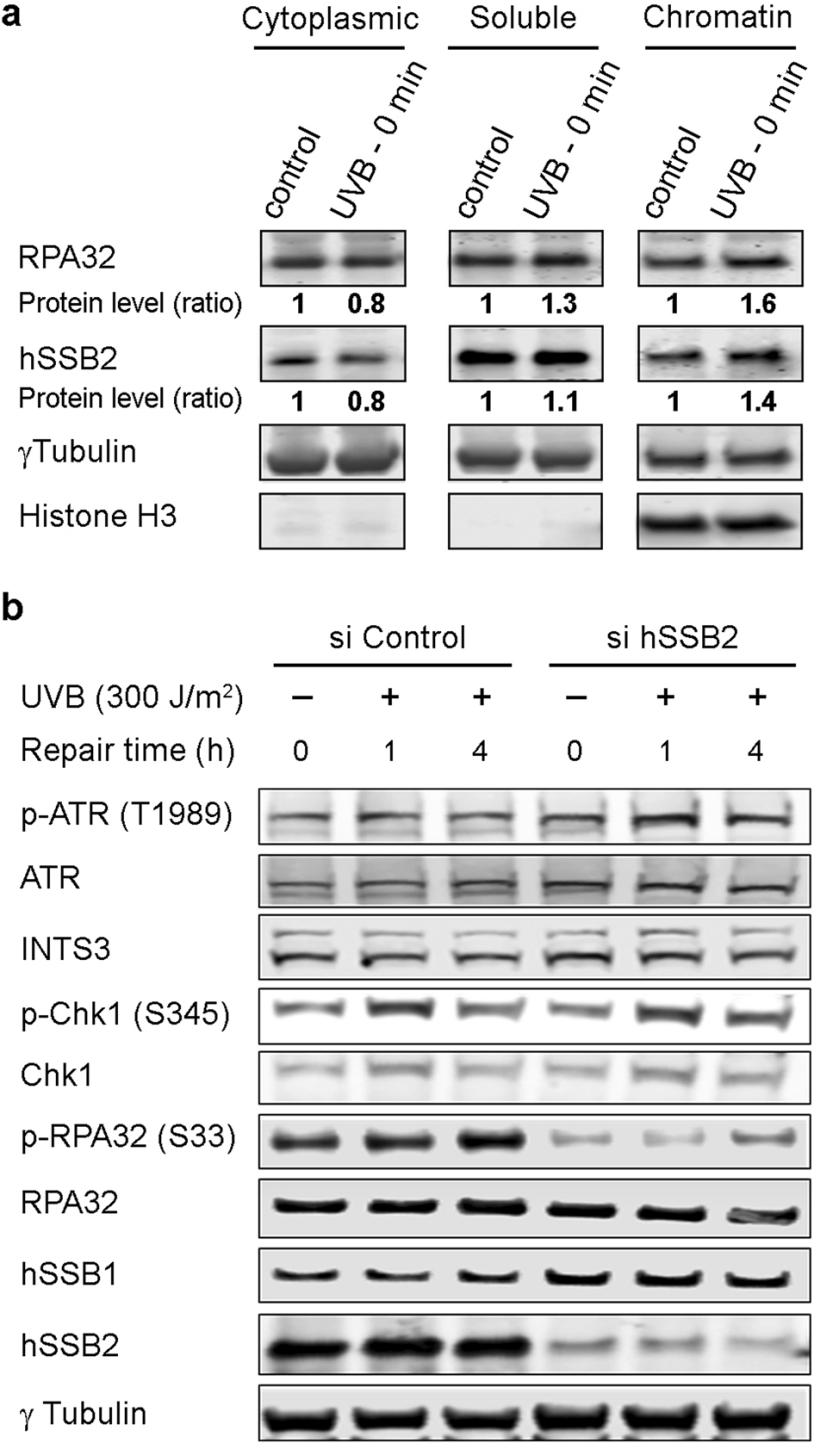
hSSB2 depletion does not affect signalling after UVB irradiation but reduces phosphorylation of RPA. (**a**) Cytoplasmic, soluble and chromatin fractions of protein extracts from HeLa cells control un-irradiated and straight after 300 J/m^2^ UVB exposure (0 min) were probed for the proteins hSSB2, RPA32, γTubulin and histone H3. Ratios of RPA and hSSB2 protein levels to control sample are indicated below the corresponding blot. γTubulin was used to normalise hSSB2 and RPA levels for cytoplasmic and soluble fractions. Histone H3 was used to normalise hSSB2 and RPA levels for chromatin fractions. Blots are representative of 3 independent experiments. (**b**) Representative western blot over 3 experiments for HeLa cells irradiated with 300 J/m^2^ UVB 48 h after transfection with control and hSSB2 siRNAs. Cells have been collected 1 and 4 h after irradiation. Blots have been probed with indicated antibody, γ-Tubulin is used as a loading control.

As we have demonstrated that hSSB2 protein is stabilized after UVB exposure in vitro and in vivo, we next sought to determine if hSSB2 binds to DNA after induction of UV damage. HeLa cells were exposed to UVB source and collected straight after irradiation. We harvested in parallel un-irradiated cells as control. Protein fractionation of cell pellets was performed and cytoplasm, nuclear soluble and chromatin bound protein fractions were collected and analysed by western blot (Fig. 2a). We observed an increase of hSSB2 level in the chromatin bound fractions immediately after irradiation compared to un-irradiated samples. This increase is reproducible and statistically significant (Supplementary Figure 2a). We also probed for RPA32 as a control^26^. In contrast to RPA32, chromatin-bound hSSB2 levels were observed to decrease 1 h and 3 h after UVB exposure (Supplementary Figure 2b). This suggests that hSSB2 rapidly and transiently binds to chromatin after UV DNA damage induction.

### hSSB2 depletion results in defective RPA32 phosphorylation after UV damage

As hSSB2 is stabilized after UVB exposure, we next determined if its depletion could impact the cellular response to UVB damage. After UVB radiation, RPA32 becomes phosphorylated by the ATR kinase on Serine 33^17^. To determine if signalling to RPA through ATR has been impacted, we analysed the UVB responsive ATR signalling pathway in control and hSSB2 depleted cells. HeLa cells were harvested at 1 h and 4 h post UVB exposure and analysed by western blot (Fig. 2b). We observed a striking reduction of RPA32 phosphorylation on Serine 33 in absence of hSSB2, while total RPA32 protein level was not affected by hSSB2 depletion. When RPA phosphorylation was defective in hSSB2 depleted cells, the ATR kinase remained active, as determined by ATR auto-phosphorylation on T1989 and ATR phosphorylation of Chk1 on Serine 345. Indeed, ATR activity in hSSB2 depleted cells was enhanced over control siRNA treated cells. As reported previously^34,47^, we also observed an increased level of hSSB1 in response to hSSB2 depletion.

### hSSB2 depletion delays XPC recruitment and impairs RPA recruitment to UV damage sites

**Figure 3:**
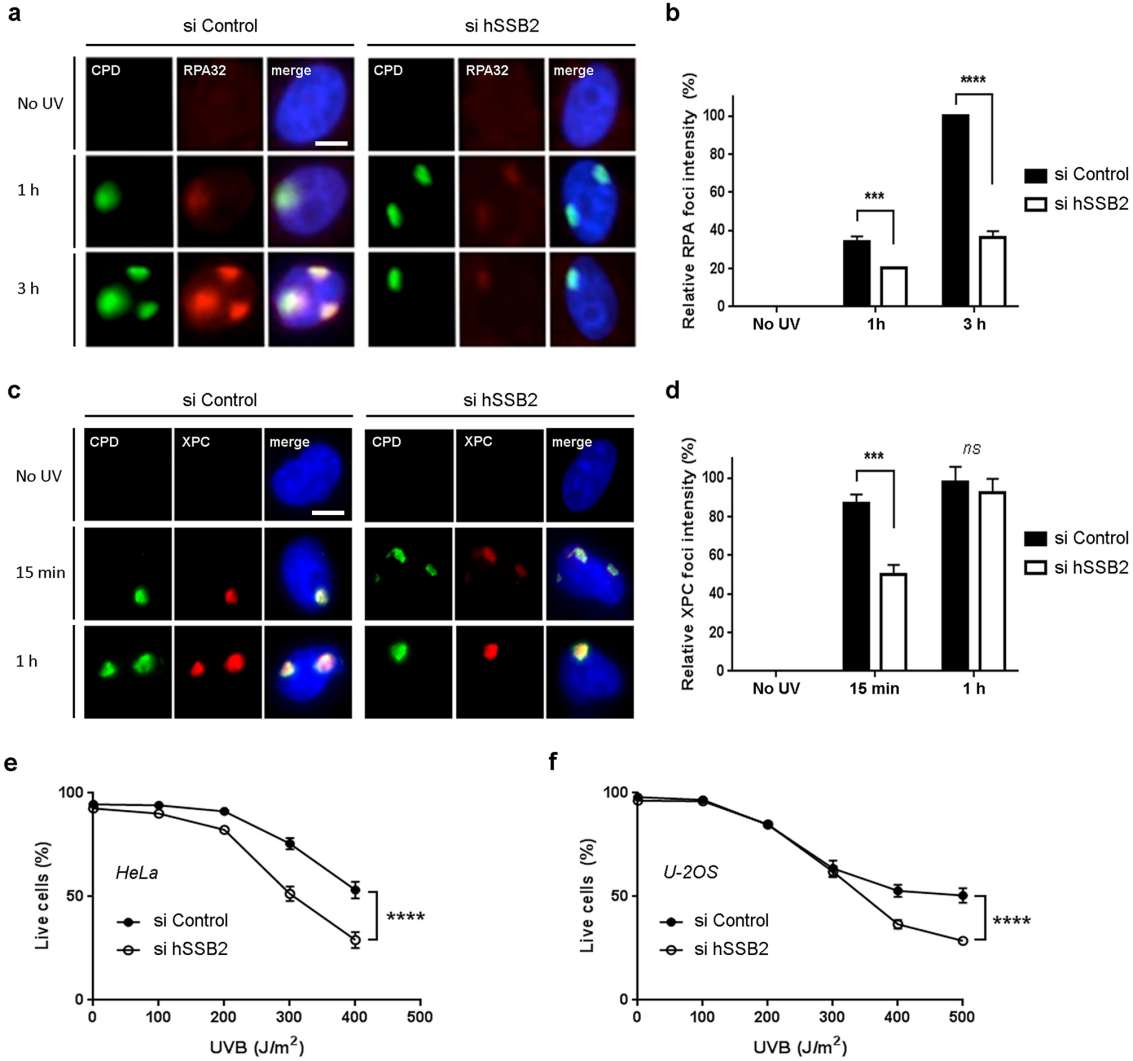
hSSB2 depletion delays XPC recruitment, impairs RPA recruitment to UV damage sites and increases sensitivity to UVB exposure. (**a**) Representative images of HeLa cells probed for CPD (green, left panels) and RPA32 (red, middle panels) without UV exposure or 1 h and 3 h after UVC irradiation with a 5 µm filter. Cells were irradiated 48 h after negative control (si Control) and hSSB2 (si hSSB2) siRNAs transfection with UVC covered with 5 µm filter. DNA is stained with DAPI (blue, right panels). Average RPA32 fluorescent staining intensity at the CPD sites (± sem, n = 3) has been reported in graph (**b**). (**c**) Representative images of HeLa cells probed for CPD (green, left panels) and XPC (red, middle panels) without UV exposure or 15 min and 1 h after UVC irradiation. DNA is stained with DAPI (blue, right panels). Average XPC fluorescent staining intensity at the CPD sites (± sem) has been reported in graph (**d**). Scale bar represents 5 µm. (**e**,**f**) Measure of the average live cells (± sem, n = 3) 48 h after UVB exposure of HeLa cells transfected with negative control (si Control) or hSSB2 (si hSSB2) siRNAs (**e**) and U-2OS cells (**f**). Analysis was carried out with the Cytell imaging system 20 min after Hoechst and Propidium Iodide staining (****P-value < 0.0001 in a Student test).

To understand the defect of RPA32 phosphorylation in absence of hSSB2, we next determined if hSSB2 was necessary for RPA32 recruitment after UVB exposure. To study the NER repair pathway, we used the UV micropore irradiation technique^52^. Briefly, HeLa cells were irradiated with a UVC source through a 5 µm pore filter. UVC light can only pass through the filter pores and thus nuclear foci of UVC DNA damage can be observed by direct and indirect markers. In control conditions, we observed an increase of RPA32 staining to the sites of UV DNA damage (marked by CPD staining) within 1 to 3 h (Fig. 3a,b, left panels), marking the normal recruitment of the RPA32 protein to the sites of induced DNA damage. However, we observed that depletion of hSSB2 by siRNA transfection resulted in a striking reduction of RPA32 staining to CPD sites following UVB treatment, as compared to control samples (Fig. 3a,b, right panels). This result suggests that hSSB2 depletion leads to a defective RPA32 recruitment to the DNA damage sites. To determine if hSSB2 directly recruits RPA32 to the site of DNA UV damage, we performed the immunoprecipitation of RPA32 with hSSB2 (Supplementary Figure 3). However, we could not determine any direct interaction between the two proteins.

We next investigated if hSSB2 depletion could also affect the recruitment of other proteins involved in NER, and more specifically upstream proteins. XPC is required for RPA recruitment to the sites of UV damage^29^. When monitoring XPC recruitment by immunofluorescence staining at CPD sites, we observed a delay in XPC level increase over time, as compared to control siRNA transfected cells (Fig. 3c,d). The delay in XPC and the failure of RPA recruitment suggests that hSSB2 is involved in the early stages of NER, and this is supported by our observed rapid binding of hSSB2 to chromatin after UVB exposure.

### hSSB2 depletion affects cell survival following UVB exposure

As RPA is required for the repair of CPDs and (6–4) photoproducts^53,54^ we next sought to determine the sensitivity of hSSB2 depleted cells to UVB radiation. For this we irradiated HeLa cells (p53 mutated) and U-2OS cells (p53 wild type) with UVB. We analysed cell viability 48 h post irradiation using a Cytell (GE) using Hoechst (live and dead cells) and Propidium Iodide (dead cells) staining (Supplementary Figure 4). Survival was automatically calculated using the Cytell software and percentage is reported in Fig. 3e for HeLa cells and in Fig. 3f for U-2OS cells. We observed that hSSB2 depletion enhances cells sensitivity to UVB irradiation independently of p53 status.

These data together with the results from above, strongly support a critical role for hSSB2 in the repair of DNA lesions generated by UVB irradiation.

### hSSB2 is a functional monomer in solution and binds ssDNA with a footprint of 5–6 nucleotides

To understand the molecular details of hSSB2 in the NER repair pathway, we next carried out biochemical and biophysical experiments with the DNA binding domain of hSSB2 (hSSB2-OB).

**Figure 4:**
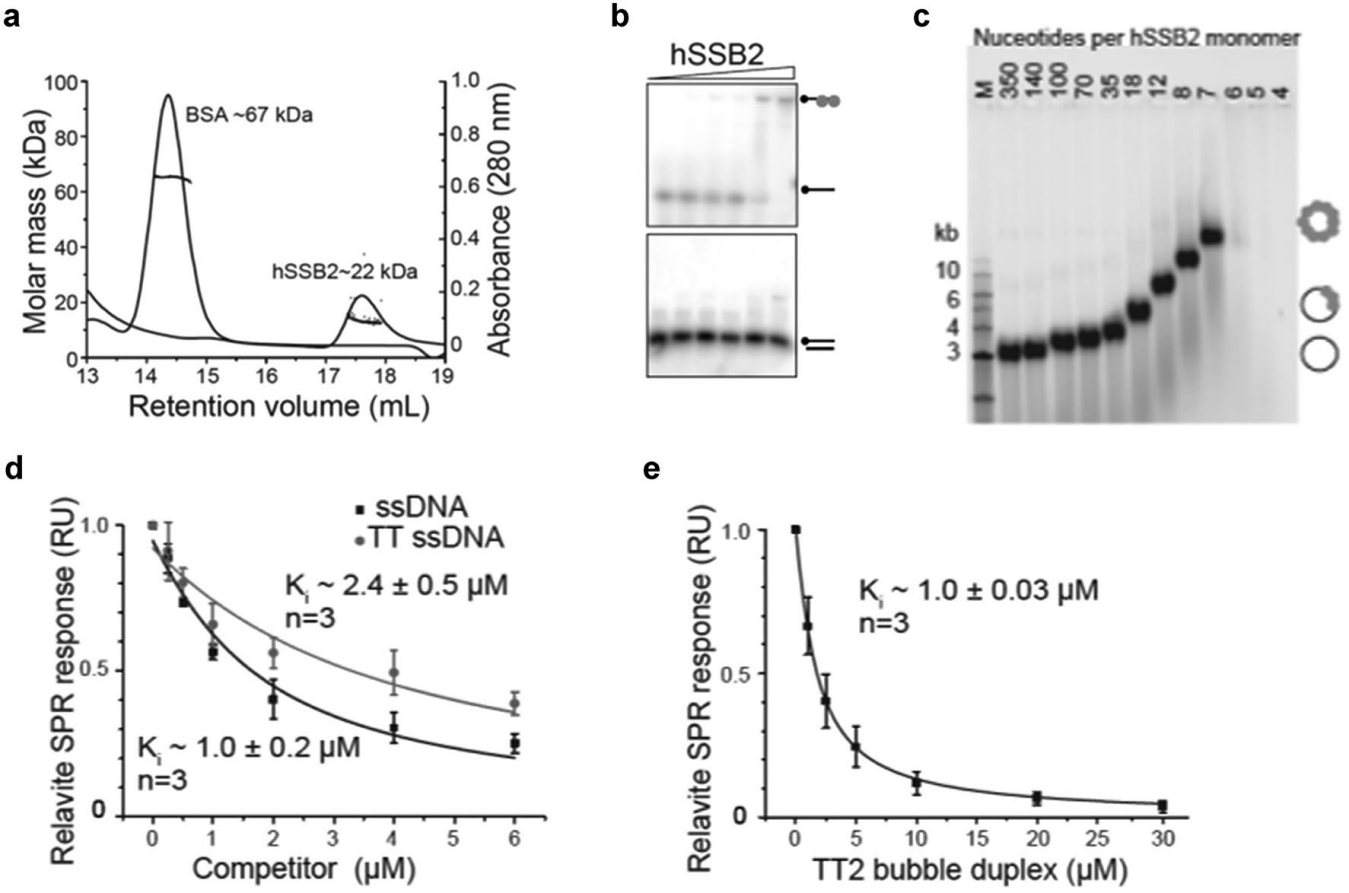
Analysis of hSSB2 binding footprint and interaction with CPDs. (**a**) hSSB2 forms monomers in a reducing solution (mimicking the normal reduced state of the nucleus). SEC-MALS spectrum of hSSB2-OB protein (~ 250 μg) that was applied to a Superose 12 column with an in line MALLS detector to determine weight-averaged molecular weight in solution. The elution profile (continuous line) and light-scattering (filled black square) are shown. Void volume (7.89 mL). hSSB2 is ~ 22 kDa; BSA (67 kDa) is shown as a comparison. (**b**) Electrophoretic mobility shift analysis showing binding of recombinant hSSB2 to ssDNA substrates, ssDNA (top) and dsDNA (bottom). The radiolabel on the ssDNA is marked with a black circle. hSSB2 binding to the oligonucleotide is indicated by grey circles. (**c**) Agarose gel shift analysis of hSSB2-OB (0–30 µM) binding to single-stranded circular bacteriophage DNA ϕX174. Nucleotides of DNA bound per hSSB2-OB monomer present are indicated. hSSB2-OB saturates bacteriophage ssDNA binding with a 5–6 nucleotide footprint (once bacteriophage ssDNA is saturated with hSSB2, this inhibits ethidium bromide intercalation and thus DNA staining), indicating a high-density binding mode. (**d**) hSSB2-OB binds the T-T lesion with almost the same affinity as it does normal ssDNA. SPR competition experiments where hSSB2-OB was injected along with increasing concentrations of a 6 mixed base single-stranded oligonucleotide in the presence (GATTGC) or absence (GATTGC; wild-type) of a CPD dimer (denoted as TT), respectively, onto a Biacore SA chip immobilized with biotinylated wild-type ssDNA. The decrease in binding of hSSB2-OB to the chip due to the inhibition from the free ssDNA was measured (inhibition constant K_i_; indicated) using the steady state equilibrium values from the binding curves. Experiments were done in triplicate (n = 3). (**e**) hSSB2-OB can recognize a 2-base stretch of non-complementary ssDNA. hSSB2-OB is flowed over the immobilised wild-type ssDNA in the presence of increasing amounts of a double-stranded oligonucleotide with a 2 nucleotide ‘bubble’ in the centre. Experiments were done in triplicate (n = 3).

SSBs from the OB domain family have been found to predominantly occur as tetramers (bacteria) or heterotrimers (eukaryotic RPA and euryarchaeota^55^) in solution. To first determine the oligomeric state of hSSB2-OB in solution (in conditions mimicking the normal reduced state of the nucleus), we performed size-exclusion chromatography coupled to multi-angle laser light scattering (SEC–MALLS) (Fig. 4a). We observed peak of absorbance for hSSB2 for a ~ 17.6 ml retention volume and matching a ~ 22 kDa mass. These data revealed that hSSB2-OB exists as a monomer, even at high concentrations. Next, to demonstrate how the OB domain of hSSB2 binds ssDNA we carried out acrylamide gel shift experiments in the presence of 32P-labelled oligonucleotides (Fig. 4b). We observed a shift in the migration of the radiolabelled ssDNA with increasing concentration of recombinant hSSB2, due to the increased binding of the protein to the DNA. These data confirmed that the protein binds single-stranded DNA (ssDNA) in analogy to its human homologue hSSB1 while displaying no affinity to double-stranded DNA^42^. To determine the ssDNA binding footprint, we performed DNA agarose gel shift experiments using the OB domain of hSSB2 and single-stranded circular bacteriophage DNA (Fig. 4c). The concentration of hSSB2-OB (expressed as a ratio of DNA nucleotide per hSSB2 monomer) is used to determine the DNA binding footprint on the circular bacteriophage DNA. We observed a reduced migration of bacteriophage DNA at 18 to 7 nucleotides per hSSB2 monomer compared to 350 nucleotides per hSSB2 monomer, showing an increased binding of hSSB2-OB to the circular bacteriophage. No bacteriophage DNA migrated through the gel at 6 to 4 nucleotides per hSSB2 monomer. hSSB2-OB recognizes ssDNA with a 5–6 nucleotide footprint (consistent with saturation of the bacteriophage DNA that inhibits ethidium bromide intercalation and thus DNA staining) indicating that this high-density binding mode is conserved between the hSSB1, hSSB2 and the crenarchaeal SSB from *Sulfolobus solfataricus* (SsoSSB). We have already used Surface Plasmon Resonance (SPR) to determine ssDNA binding stoichiometry and affinity of hSSB2 in another study^56^, revealing a 1:1 binding mode and a dissociation constant of 1.3 µM.

### hSSB2 has comparable affinity for CPD dimers and 2-base stretches of ssDNA

We next sought to determine whether hSSB2 is able to directly bind to T-T lesions. Surface plasma resonance (SPR) is a surface based technology commonly used for studying protein DNA interactions and provides detailed kinetic information about the protein DNA interaction. Using SPR competition experiments as previously described^56^, in which we injected hSSB2-OB protein as well as increasing concentrations of a 6 mixed base single-stranded oligonucleotide in the presence (GATTGC) or absence (GATTGC; wild-type) of a CPD dimer (denoted as TT), respectively, onto a Biacore SA chip that bore immobilized biotinylated wild-type ssDNA (Fig. 4d). This experiment sought to determine if the binding affinity of hSSB2 to a ssDNA substrate containing a damaged T-T crosslink was similar to undamaged ssDNA. Surprisingly, our data revealed that hSSB2-OB binds the T-T lesion with almost the same affinity as normal ssDNA, indicating that the protein is capable of binding both damaged (CPD dimers) and undamaged single strands of a damaged duplex. The next stage was to determine if hSSB2 had the ability to bind to duplex DNA that contained a two-base mismatch mimicking a CPD moiety. This is important in the context of damaged duplex DNA within the cell as this will be present within a duplex structure. To confirm that hSSB2-OB is indeed able to recognize a 2-base stretch of non-complementary dsDNA we carried out a similar set of SPR competition experiments using hSSB2-OB in the presence of increasing amounts of a 23mer double-stranded oligonucleotide in which two adenine bases in the centre of the complementary strand were substituted by cytosines (creating a 2-base ‘bubble’) (Fig. 4e). Importantly, our SPR data revealed a binding affinity of 1.0 µM, which is equivalent to the binding to ssDNA (Fig. 4e and^57^) indicating that the OB domain of hSSB2 can recognize and bind very short stretches of ssDNA that are formed within a DNA duplex at CPD-damaged sites.

## Discussion

In this study, we sought to understand the role of the SSB family member hSSB2 in the response of human cells to UV radiation. The data presented here, specifically implicate hSSB2 in the NER repair pathway. While there have been a number of studies describing the role of hSSB1 in DNA repair, the role of hSSB2 within the cell has proven more elusive, with only its function in DNA double-strand break repair described thus far^33,34^. hSSB1 and hSSB2 have highly homologous OB fold domains but their tails diverge, raising the possibility that they each have unique cellular functions. Indeed, hSSB1 is ubiquitously expressed in cells, while hSSB2 is restricted to only a few tissue types (notably thymus, spleen, testis and skin^58,59^) and cancer cell lines^59^. Even if hSSB1 and hSSB2 are likely to have differing cellular functions, there is evidence that they have the capacity to compensate for the loss of each other in specific pathways in mice^47,48^.

In the current study, we report the role of hSSB2 in the response to UVB induced DNA damage. We observed an increase of hSSB2 protein level after UVB exposure in vitro and in vivo. hSSB1 is also known to be rapidly stabilized following the induction of double strand DNA breaks^31,33,34^, with stabilization being dependent on hSSB1 phosphorylation on threonine 117 by the ATM kinase. hSSB2, however, lacks any ATM or ATR SQ/TQ consensus sequences suggesting its upregulation is not mediated directly by these kinases.

It is known that UV radiation induces RPA32 phosphorylation^60^. In our current study, we show that the absence of hSSB2 leads to a significant decrease of RPA32 phosphorylation (another member of the SSB protein family) after UV exposure (Fig. 2). We suggest that this defect is due to a significant reduction in RPA32 recruitment to the sites of DNA damage, which likely impedes its ATR dependent phosphorylation (Fig. 3a,b). Dissecting the potential involvement of hSSB2 in replication stress and the molecular detail of hSSB2 localisation to chromatin and the dispersal from chromatin after UVB irradiation will require further study. However, we were not able to detect hSSB2 by standard immuno-fluorescence staining following UVB in situ (data not shown). The rapid transient localisation of hSSB2 to the damage site could be shorter than the time needed to process the samples at the end of UVB irradiation.

We also observed that hSSB2 depletion delays the recruitment of XPC (Fig. 3c,d), but unlike with RPA32, a comparable level of XPC to control cells is eventually recruited to the site of damage, suggesting a minor role of hSSB2 in damage recognition. Moreover, we did not observe any interaction between hSSB2 and XPC by immunoprecipitation at the timepoints we examined (Supplementary Figure 5). However, we consider it possible that hSSB2 may have an indirect role to stabilise the opening of the chromatin at the site of the UV DNA damage to promote the recruitment of other repair factors.

For this study, we performed experiments on 80% confluent cells with a low population of cells in S phase. We thus focused on NER as this pathway is independent of DNA replication, however we can not exclude that some of the UV damage induced in our experiments was repaired by other repair pathways. During DNA synthesis, other specific DNA repair pathways can intervene^61^. Translesion synthesis (TLS) allows the replication of UV damaged DNA by an error-prone DNA polymerase^62^. Unrepaired UV DNA damage can also inhibit the progression of DNA replication forks, potentially leading to the formation of a DNA double-strand break which requires homologous recombination (HR) for repair^63^. The mismatch repair pathway (MMR) has also been reported to repair a subset of UV-induced DNA damage^64,65^. The HR and MMR pathways are not specific to the repair of UV-induced DNA damage, but significantly they also both involve the RPA complex, therefore it is likely that the inhibition of hSSB2-dependent RPA recruitment could also disrupt these repair pathways. hSSB2 involvement in HR has been previously reported^46^ and further work is required to determine if hSSB2 depletion affects the TLS or MMR pathways and the repair of DNA replication forks.

To understand the molecular details of hSSB2 in this process, we next focused on the DNA binding properties of hSSB2 (Fig. 4). Our data reveal that hSSB2 is capable of binding ssDNA that contains CPD lesions with similar affinity to normal unchanged ssDNA oligos. Moreover, we demonstrate hSSB2 interaction with a double-stranded DNA (dsDNA) with non-two complementary bases in the centre, indicating that hSSB2 is able to open up UV-damaged dsDNA. However, it is likely that hSSB2 binds to a larger sequence that transiently becomes available by destabilization of nucleotides on either side of the mismatch.

The binding of hSSB2 to the site of damage promotes the recruitment of the heterotrimeric RPA complex. It is still unclear how RPA is recruited as we did not observe a direct interaction between hSSB2 and RPA32. This raises the question of why two SSB proteins are required in the same process. It can be speculated that hSSB2 can bind with high affinity to short 5–6 nt DNA sequences, while RPA has a preference for much longer sequences of 35 nt and above. It has previously been shown in vitro that RPA binding to DNA triggers checkpoint signalling^66,67^. However, those in vitro systems do not present the complexity of genomic DNA, notably chromatin compaction within nucleosomes, reducing the access of repair proteins to damage sites. This suggests a model whereby hSSB2, with a smaller DNA footprint, may bind a small region of DNA containing UV-induced adducts, promoting the disruption of dsDNA to produce long stretches of ssDNA, to which the RPA complex may then bind.

Previously, it has been reported that recruitment of the RPA complex to the DNA damage site through ATRIP interaction is required for the activation of ATR kinase activity^26^. Since RPA recruitment is defective in hSSB2 deficient cells, we also expected to see a defect in the phosphorylation of ATR targets. Consistent with this, we observed that RPA phosphorylation was abrogated in cells depleted of hSSB2, however, we did not observe a defect in Chk1 phosphorylation (Fig. 2). This suggests that the ATR signalling pathway is not completely disrupted by hSSB2 depletion and that UV-induced ATR kinase activity may not be completely dependent upon the recruitment of the RPA complex. Kar et al.^40^, reported previously that hSSB1 could promote ATR-dependent signalling in the absence of the RPA complex. Our data also support this observation, and it is possible that hSSB1, upregulated by hSSB2 depletion (as observed in Fig. 2), could promote ATR signalling pathway in the absence of RPA.

Further studies will be required to determine why hSSB2 loss cannot be compensated by hSSB1 or other proteins for the recruitment of RPA to the site of DNA damage, but RPA loss can be compensated to initiate the DNA damage signalling response.

We conclude that hSSB2 is involved in the response to UVB induced DNA damage to promote the recruitment of RPA. In summary this study has uncovered a new cellular role for the single-stranded DNA binding protein hSSB2 in the response to UVB induced DNA lesions. This highlights the importance of the simple SSB family in maintaining genomic stability.

## Material and methods

### Cell culture

HeLa and U-2OS cells were maintained in Roswell Park Memorial Institute medium (RPMI, Sigma) and α–MEM (Gibco) respectively. All cell culture media was supplemented with 10% foetal bovine serum (Sigma). Cells were kept in culture at 37 °C in humidified atmosphere and 5% CO_2_.

### Expression constructs, siRNA and transfections

Stealth siRNA against hSSB2/NABP1 were synthesized by Invitrogen (Life Technologies) HSS148948. Individual siRNA sequences were 5′-GAAUAGUAAUAUGGGUACAGGUACA-3′ (sense) and 5′-UGUACCUGUACCCAUAUUACUAUUC-3′ (antisense). Controls for off target transfections were performed using Stealth Negative Control siRNA (12935300, Life Technologies). siRNAs were transfected using RNAiMax (Life Technologies) in OptiMEM (Gibco).

### UV irradiation

Cells were grown on 24 well plates. Ultra Violet B irradiations were carried on with a UVB Phillips bulb (TLW20W-01RS). HeLa cells culture media was replaced by Phosphate Buffer Saline (PBS) for the time of UV exposure. At the end of the repair time, culture media was removed and cells fixed with 4% paraformaldehyde (Electron Microscopy Sciences, Fischer Scientific) in PBS (for 20 min, room temperature). Samples were then kept in PBS at 4 °C.

For UVC irradiations experiments, cells were grown on sterile glass coverslips (ProSciTech, Kirwan, Australia), in a 6 cm diameter dish. Cells were washed once with PBS. Once PBS was discarded, a 5 µm filter (Milipore) was put on top of the cell monolayer just before UVC exposure (260 nm, Bio-Link BLX-254). After exposure to UV irradiation, filter was removed and cells were put back in culture medium for recovery. At the end of the repair time, cells were incubated for 5 min with ice-cold pre-extraction buffer (20 mM Hepes, 20 mM NaCl, 5 mM MgCl_2_, 0.5% IGEPAL) before being fixed following the protocol described above.

### Cell sensitivity

To measure cell sensitivity, HeLa cells were seeded in a 24 well plates (Costar) and incubated for 48 h after UVB exposure. Hoechst dye (1 µg/ml) and Propidium Iodide (PI, 10 µg/ml) were added to culture medium for 20 min (37 °C) before analysing the plate with the Cytell automated system (GE Healthcare). Ratio of dead cells (PI positive) is calculated by using the cell viability imaging software (Cytell software, GE Healthcare).

### Antibodies

The antibodies against ATR (2790), Chk1 (2345), Ser345 phospho-Chk1 (2341), RPA32 (52448), XPC for immuno-precipitation assay (14768) and Histone H3 (9715) were purchased from Cell Signaling. IntS3 (A302-051A) and Ser33 phospho-RPA32 (A300-246A) antibodies were purchased from Bethyl Laboratories. Antibodies against XPC for immuno-fluorescence assay (GTX70308) and Ser1989 phospho-ATR (GTX128145) were purchased from Genetex. CPD (T1192) and gamma-tubulin (T6557) antibodies were purchased from Sigma. Antibodies against histone H2B (ab1790) and hSSB2 (16419-1-AP) were purchased respectively from Abcam and ProteinTech. The hSSB1 antibody was purified from sheep anti-serum as described previously^31^. Fluorescent secondary antibodies used were: Donkey anti-Mouse 800 nm (LI-COR; IRDye 800CW 926–32,212, 1:5000 for WB), Donkey anti-Rabbit (LI-COR; IRDye 680LT 926–28,023, 1:5000 for WB) and Alexa Fluor 488 (A32766, Molecular Probes, 1:200 for IF) and 594 (A32754, Molecular Probes, 1:200 for IF).

### Western blotting

Cells were grown in 10 cm dishes to confluent density. After UVB irradiation and incubation from 0 to 4 h, whole cell proteins were extracted as previously described^35^. Briefly, cells were lysed (20 mM Hepes pH 8.0, 150 mM KCl, 5% glycerol, 10 mM MgCl_2_, 0.5 mM EDTA, 0.02% NP-40, freshly supplemented with NaF, NaVO_4_, PMSF and protease inhibitors—Roche) and sonicated. Lysates were cleared by centrifugation and protein concentrations were estimated using the BCA assay (ThermoFisher Scientific). 25 μg of total cell lysates was resolved by 12% SDS-PAGE followed by transfer onto a nitrocellulose membrane. Membranes were blocked in 2% fish skin gelatin in PBS (Sigma) for 1 h and incubated with primary antibodies overnight 4 °C in Odyssey PBS Blocking Buffer (LI-COR). Following incubation with IR Dye secondary antibodies (LI-COR), membranes were visualised using a LI-COR Odyssey infrared scanner. Intensity analysis has been performed with Image Studio Lite software (LI-COR) and statistical analysis was generated with Prism software (GraphPad).

### Immunohistochemistry

Mouse skin samples were irradiated with a UVB source and collected as described previously^51^. Briefly, pups (3 days old) were exposed to a single UVB exposure from a bank of six Phillips TL100W 12RS UVB lamps (total UVB = 5.9 kJ/m^2^). Skin was collected different time after irradiation and fixed in formalin 10% for 24 h and kept in ethanol 70%. Skin sections were stained for SSB2 (with Ssb2−/− mouse skin used as a negative control for labelling specificity) following the protocol described previously^47^ and for CPD following provider’s recommendations (Sigma-Aldrich).

### Protein fractionation

Protein fractions of HeLa cells after exposure to UVB radiation for cytoplasmic, nuclear soluble and chromatin-bound fractions were generated using the Subcellular Protein Fractionation Kit (ThermoFisher Scientific). Fractions obtained following manufacturer instruction from fresh cell pellets were kept at − 30 °C until western blot analysis.

### Immunofluorescence

Fixed cells were incubated 10 min on ice with 0.2% Triton X-100 (Sigma) in PBS (Triton buffer) and blocked in PBS with 3% BSA (Sigma) for 1 h. For CPD staining, an extra step for DNA denaturation was added with 1 N HCl for 5 min and then washed 3 times with PBS. Cells were incubated with the primary and secondary antibodies for 1 h each at room temperature, followed by a 5 min incubation with DAPI (1 µg/ml in PBS) , and washed with PBS. The coverslips were mounted on a slide with Prolong Gold. Images were acquired with a Deltavision system (GE Healthcare) and analysed with ImageJ software. Intensity of fluorescence of XPC and RPA32 proteins to CDP foci was measured using CDP staining as a mask that allows the measure specifically the staining in the DNA damage region of interest. A minimum of 50 cells per sample were analysed, with experiments for each staining performed independently 3 times.

### Immuno-precipitation

Pull down of hSSB2 and XPC: the cells were harvested and lysed in a buffer (20 mM HEPES pH 8.0, 150 mM KCl, 1 mM EDTA pH 8.0, 0.05% Igepal, 1× Phosphatase Inhibitor Cocktail and 1× of phosphatase cocktail) using sonication. Afterwards, the samples were spun at 16,000*g* at 4 °C for 10 min and supernatant were subjected for the determination of protein concentration using BCA assay (ThermoFisher Scientific). Protein G dyna beads (ThermoFisher Scientific) were prepared by incubating it with 2 µg of anti-OBFC2A (ProteinTech) or XPC antibody (Cell Signaling) for 30 min at room temperature. Later on, beads were washed 3 times with IP buffer and cell lysates were incubated with prepared protein G dyna beads for 90 min at 4 °C. After incubation, the supernatant was aspirated, and beads were washed 6 times with a lysis buffer. Proteins were eluted from the beads with 2× SDS loading dye and run on 4–12% SDS-PAGE gradient gel for western blot.

## Protein purification

The DNA-binding domain of hSSB2 (hSSB2-OB, residues 1–132) was expressed and purified as described previously^57^. Briefly, the protein was expressed in *Escherichia coli* BL21(DE3) from a pGEX-6P vector as a GST-fusion protein at 37 °C under standard conditions; hSSB2-OB was purified using glutathione affinity chromatography, HRV3C protease cleavage and size exclusion chromatography as the final purification step. Protein concentration was determined by absorbance using the theoretical extinction coefficient at 280 nm.

### Size exclusion chromatography-multi-angle light scattering (SEC-MALLS)

Size exclusion chromatography coupled to multi-angle laser light scattering was carried out as described previously^68^. Briefly, 250 µg of purified recombinant hSSB2-OB protein in MALLS buffer (20 mM Tris pH 7, 100 mM NaCl, 1 mM EDTA, 1 mM TCEP) was injected onto a Superose 12 10/30 analytical size exclusion column mounted to an AKTA chromatography system in tandem with a MALLS detector. Monomeric BSA was used as a reference to determine the molecular weight of the target proteins.

### Electrophoretic mobility shift assay (EMSA)

EMSA experiments have been carried out as described previously^31^. Briefly, increasing concentrations of hSSB2-OB (0– μM) were incubated with 100 fmol of ^32^P, radiolabelled oligonucleotide substrates (CCTCGAGGGATCCGTCCTAGCAAGCCGCTGCTACCGGAAGCTTC TGGACC and its complement to create the duplex GGTCCAGAAGCTTCCGGTAGCAGCGGCTTGCTAGGACGGATCCCTCGAGG) in EMSA buffer (20 mM Tris pH 7.5, 100 mM NaCl, 1 mM DTT 100 μg/mL BSA) for 15 min at 37 °C. Samples were resolved on a 10% native polyacrylamide/TBE gel, the phosphorimage visualized using a FLA-9000 image scanner (Fujifilm), and quantified using MultiGauge software (Fujifilm).

### Agarose gel shift analysis

Agarose Gel shift experiments were carried out using a range of concentrations of purified hSSB2-OB protein (0–30 µM) in phosphate buffer (10 mM phosphate pH 6.0, 50 mM NaCl, 1 mM EDTA,1 mM DTT) with 100 ng of single stranded phage phiX174 DNA and 1 mg/mL Bovine Serum Albumin in a total 10 µL volume. After incubating the reaction mixture for 20 min at 20 °C, Orange G loading dye (30% (w/v) glycerol, 0.2% (w/v) Orange G) was added to the sample and electrophoresed on a 1% TAE agarose gel at 80 V for 100 min. The gel was stained for 1 h in the dark using SYBR gold fluorescent stain diluted 1/10,000 in TAE buffer. The gel was rinsed thoroughly in MQW and scanned using a fluorescence imager (Typhoon™ FLA 9000 Biomolecular Imager).

### Surface plasmon resonance (SPR) binding studies

Competition SPR experiments have been carried out as described previously^69^. Briefly, protein samples and oligonucleotide (obtained from Sigma-Aldrich) dilutions were completed in SPR buffer (20 mM Tris pH 7.0, 50 mM NaCl, 2 mM MgCl_2_, 0.01% P20, 2 mM DTT). The competition experiments were performed by injecting hSSB2-OB protein as well as increasing concentrations of a 6mer mixed base single-stranded oligonucleotide in the presence (5′-GATTGC-3′) or absence (5′-GATTGC-3′; wild-type) of a CPD dimer (denoted as TT), respectively (Fig. 4d) or a 23mer mixed double-stranded oligonucleotide with a mismatch (indicated in bold) in the centre (5′-CGAACGATCA**TT**ACTAGCTCCAA-3′ with complementary sequence with mismatch 3′-TTGGAGCTAGT**CC**TGATCGTTCG-5′) (Fig. 4e) onto a Biacore SA chip that bore immobilized biotinylated wild-type ssDNA. As increasing ratios of protein to free DNA samples were flowed over the chip, the decrease in binding of protein to the chip due to the inhibition from the free ssDNA was measured using the binding curves generated. The inhibition constant was measured using the steady state equilibrium values from the binding curves, then fitted to the equation below to determine the inhibition constant of binding interactions.

$${R}_{equ}={k}_{prop}\times \left({P}_{T}-\left(\frac{{P}_{T}+{DNA}_{T}+{K}_{soln}}{2}\right)+\sqrt{{\left(\frac{{P}_{T}+{DNA}_{T}+{K}_{soln}}{2}\right)}^{2}-{P}_{T}\times {DNA}_{T}}\right)$$

R_equ_—equilibrium SPR response, k_prop_—proportionality factor, P_T_—total protein concentration, DNA_T_—total DNA concentration, K_soln_—dissociation constant.

### Animal ethics

The animal experiments were designed and carried out according to the Australian code for the care and use of animals for scientific purposes (the Code; Clause 2.4.34), which is in compliance with the ARRIVE guidelines. Institutional animal ethics approval from QIMR Berghofer Research Institute was obtained for the animal experimentation (A98004M)^51^.

## Supplementary Information

Supplementary Information.
