# Supplementary material for: hSSB2 (NABP1) is required for the recruitment of RPA during the cellular response to DNA UV damage
Source: Sci Rep. 2021 Oct 12;11:20256. doi: 10.1038/s41598-021-99355-0 (PMC8511049; doi:10.1038/s41598-021-99355-0)

hSSB2 (NABP1) is required for the recruitment of RPA during the cellular response to DNA UV damage

Didier Boucher[1], Ruvini Kariawasam[2], Joshua Burgess[1], Adrian Gimenez[3], Tristan E. Ocampo[3], Blake Ferguson[4], Ali Naqi[5], Graeme J. Walker[6], Emma Bolderson[1], Roland Gamsjaeger[2,3], Kenneth J. O'Byrne[1,7], Liza Cubeddu[2,3]*, Kum Kum Khanna[8]* and Derek J. Richard[1]*

1. Queensland University of Technology (QUT), Cancer & Ageing Research Program, Centre for Genomics and Personalised Health at the Translational Research Institute (TRI), Brisbane, Australia

2. School of Science and Health, Western Sydney University, Penrith, NSW 2751, Australia

3. School of Life and Environmental Sciences, University of Sydney, NSW 2006, Australia

4. Drug Discovery Group, QIMR Berghofer Medical Research Institute, Herston, QLD 4006, Australia

5. Department of Chemistry, Pennsylvania State University, University Park, USA

6. Diamantina Institute, University of Queensland, Woolloongabba, QLD 4102, Australia

7. Princess Alexandra Hospital, Woolloongabba, QLD 4102, Australia

8. Signal Transduction Group, QIMR Berghofer Medical Research Institute, Herston, QLD 4006, Australia

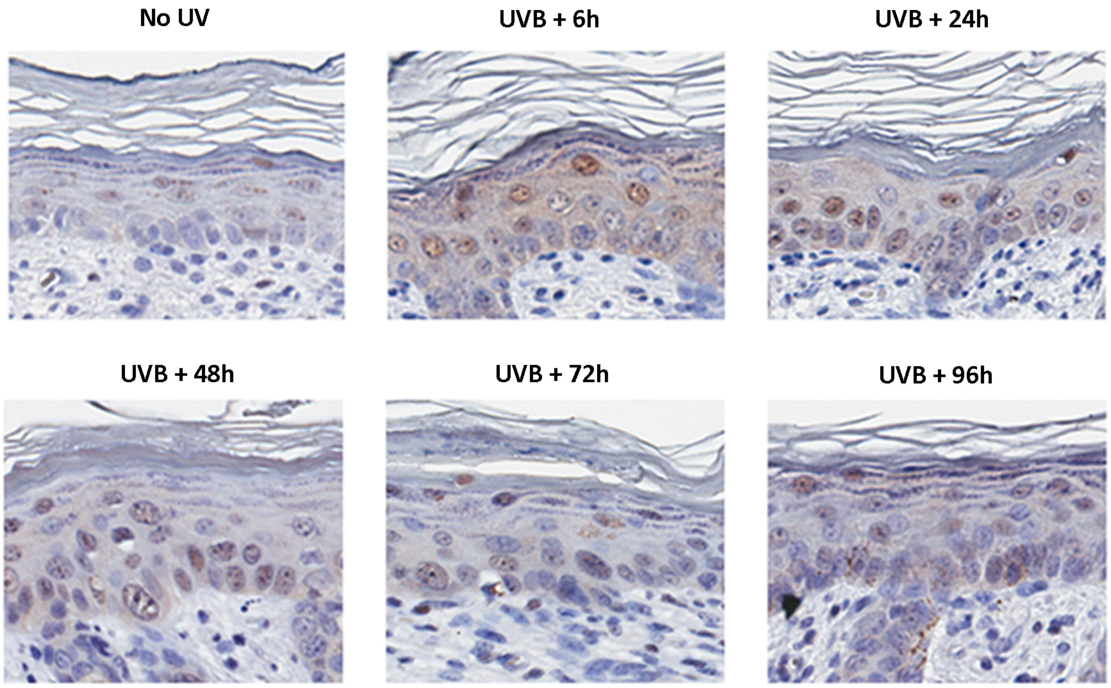

Supplementary Figure 1. SSB2 level increases in mouse keratinocytes after UV B exposure. Immuno-histo chemistry staining for SSB2 (in brown) of skin sections from 3 days old C57BL6/J mice wild type and 6, 24, 48, 72 and 96 h post UVB exposure.

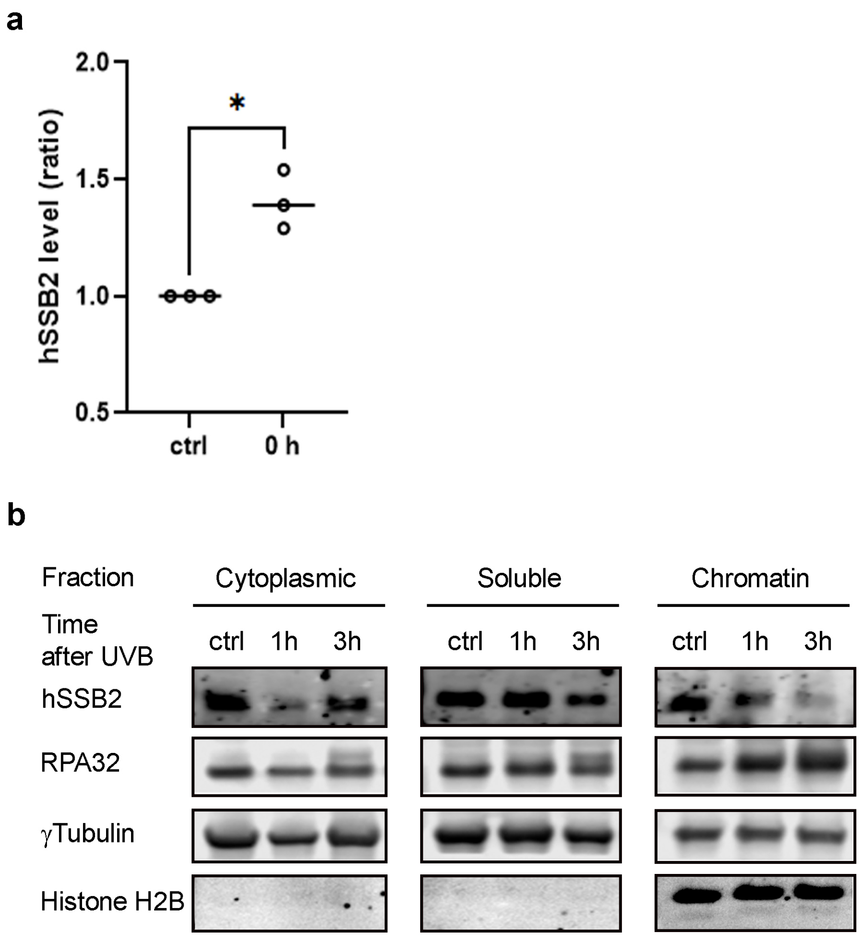

Supplementary Figure 2. Transient binding of hSSB2 to chromatin after UVB exposure.

a. Average level of hSSB2 measured by western blot in chromatin fraction of protein extracts from HeLa cells straight after 200 J/m^2 UVB exposure (0 h) versus unirradiated control (ctrl) over 3 independant experiments. hSSB2 level were normalised to histone H3. * p-value = 0.0305 in a paired t-test.

b. Cytoplasmic, soluble and chromatin fractions of protein extracts from HeLa cells collected 1 and 3 h after 200 J/m^2 UVB versus unirradiated control (ctrl) were probed for the proteins hSSB2, RPA32, γTubulin and histone H2B. Blots are representative of 3 independent experiments.

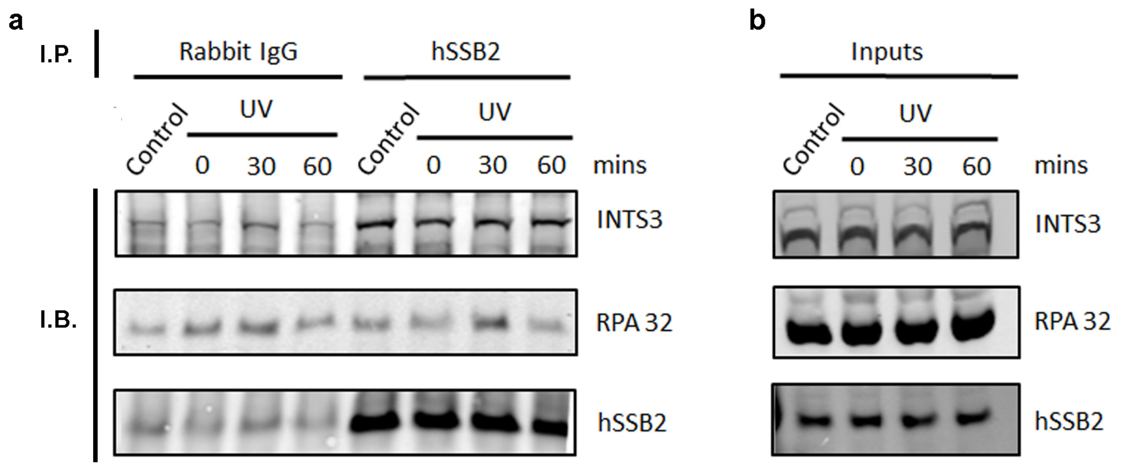

Supplementary Figure 3. Absence of direct interaction between hSSB2 and RPA32.
a. Lysates from Hela cells after the indicated incubation time post 200 J/m^2 UVB were immunoprecipitated (I.P.) with control rabbit IgG (as a negative control) or hSSB2 antibody. Immunoprecipitates were immunoblotted (I.B.) with the indicated antibodies. The protein INTS3 is used as a positive control to verify hSSB2 proper immuno-precipitation.
b. Whole cell lysates were also immunoblotted with the same antibodies for control.

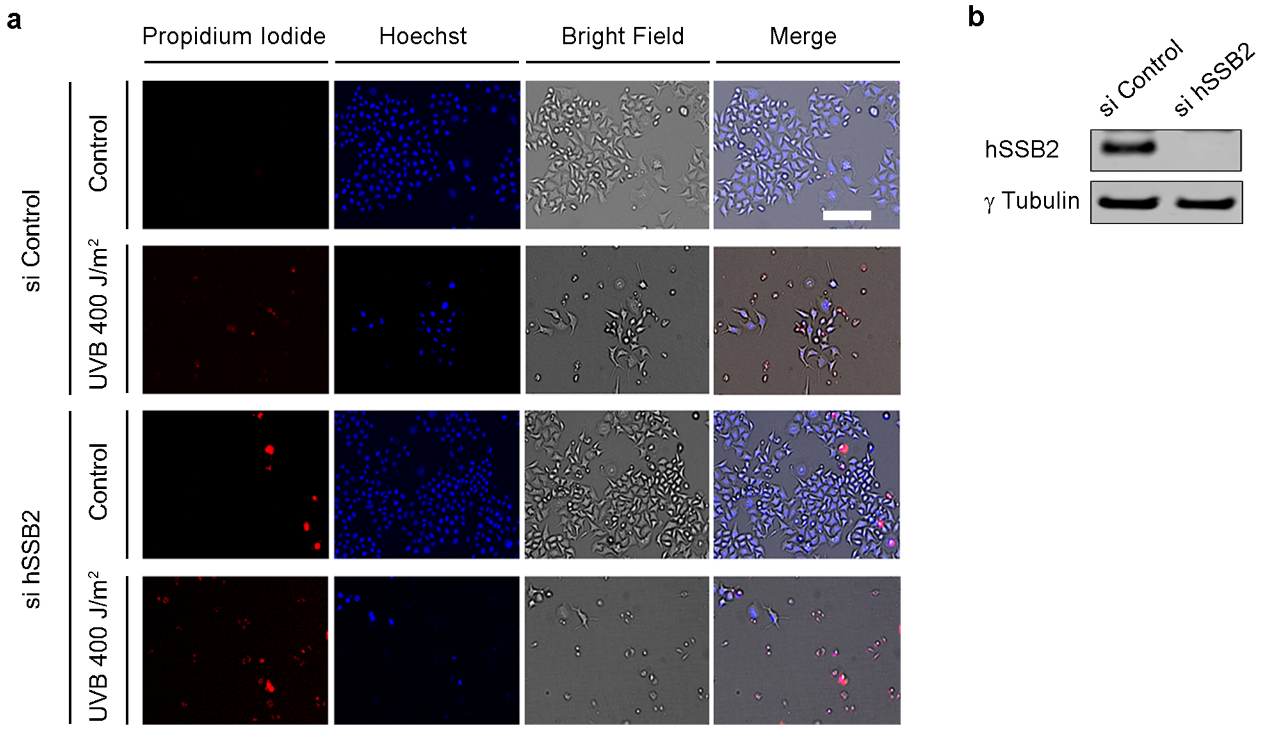

Supplementary Figure 4. Increased sensitivity of HeLa cells to UVB when depleting hSSB2.

a. Representative images over 3 independant experiments of Hoechst (blue) and Propidium Iodide (PI, red) staining of HeLa cells transfected with control (top panels) or hSSB2 siRNA (bottom panels) 48 h after exposure or not to 400 J/m^2 UVB. Images were acquired with the Cytell system (GE) to count dead cells (PI positive nuclei, red) over all cells (Hoechst stained nuclei, blue). Scale bar represents 50 μm.

b. Verification of hSSB2 depletion 48 h after siRNA transfection of HeLa cells by western blot analysis on whole cells protein extracts from samples used for survival assay (a). γTubulin is used as proteins loading control.

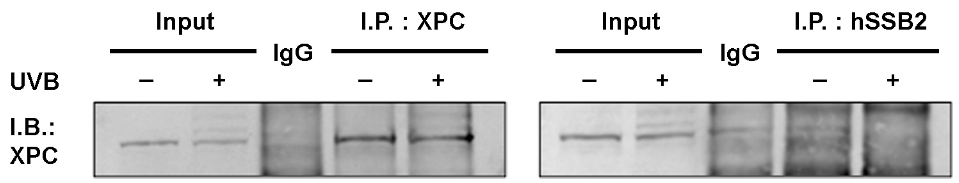

Supplementary Figure 5. Absence of direct interaction between hSSB2 and XPC. Lysates from Hela cells un-irradiated or 30 min after UVB exposure were immunoprecipitated (I.P.) with control rabbit IgG (as a negative control) or XPC (left panel) and hSSB2 (right panel) antibodies. Immunoprecipitates were immunoblotted (I.B.) with XPC antibody. Whole cell lysates were also immunoblotted for control (Input).

Supplementary figure 6: original digital images of cropped blots

Figure 1a

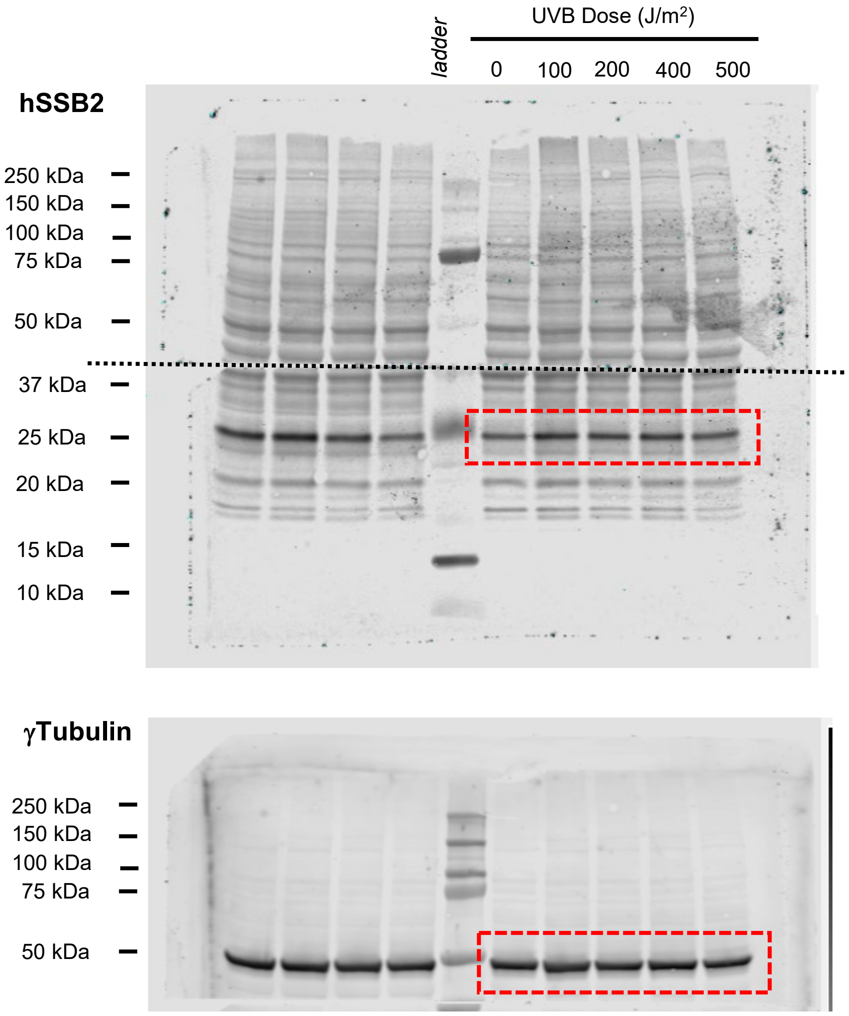

Figure 1b

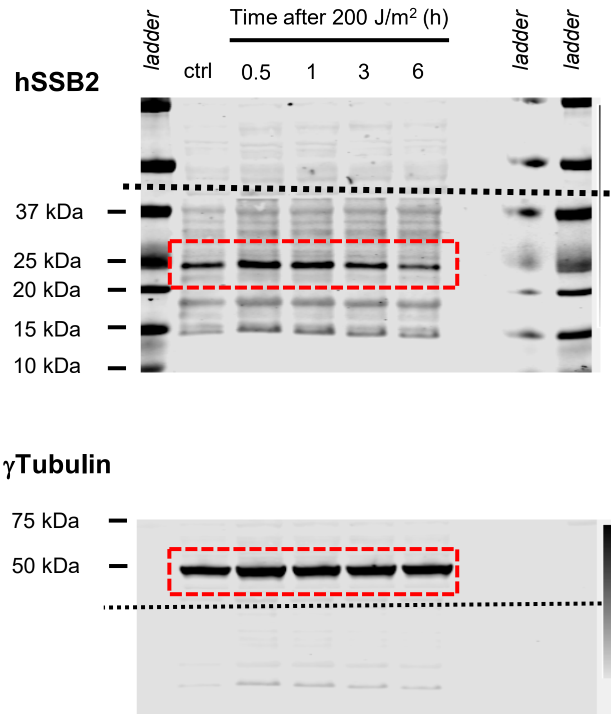

Figure 2a

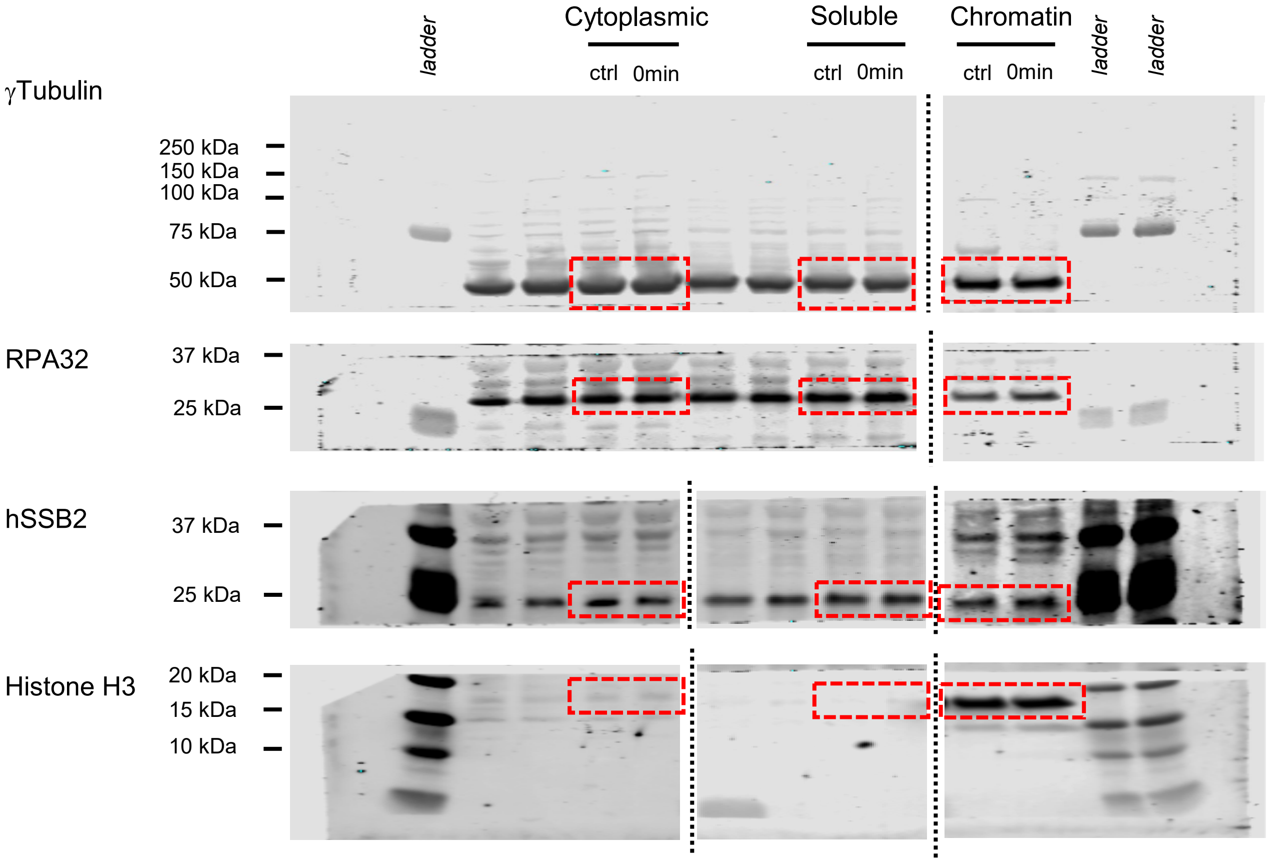

Figure 2b

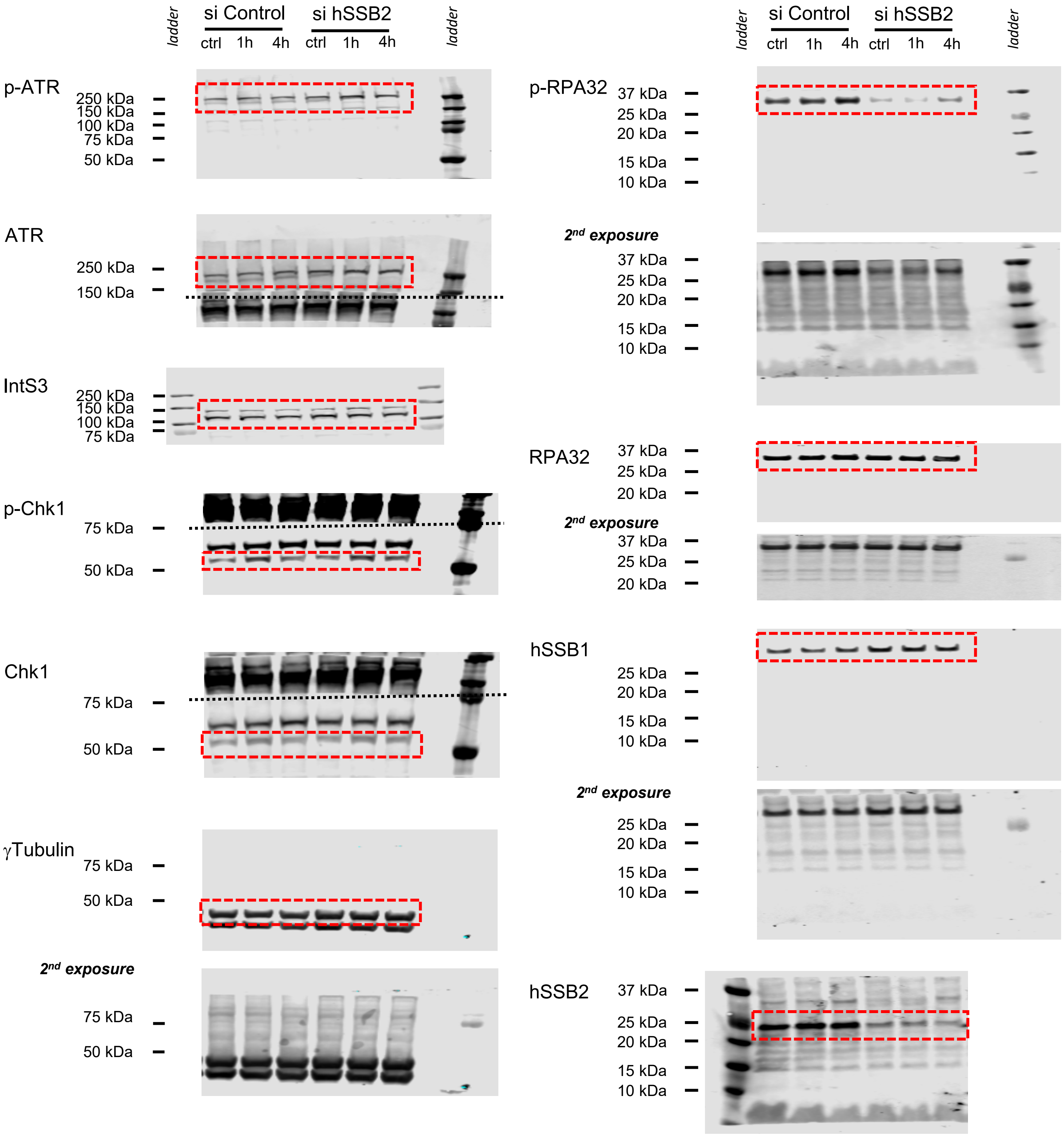

ladder
si Control
si hSSB2
ctrl
1h
4h
ctrl
1h
4h
ladder
p-ATR
250 kDa
150 kDa
100 kDa
75 kDa
50 kDa
ATR
250 kDa
150 kDa
IntS3
250 kDa
150 kDa
100 kDa
75 kDa
p-Chk1
75 kDa
50 kDa
Chk1
75 kDa
50 kDa
γTubulin
75 kDa
50 kDa
2nd exposure
75 kDa
50 kDa
p-RPA32
37 kDa
25 kDa
20 kDa
15 kDa
10 kDa
2nd exposure
37 kDa
25 kDa
20 kDa
15 kDa
10 kDa
RPA32
37 kDa
25 kDa
20 kDa
2nd exposure
37 kDa
25 kDa
20 kDa
hSSB1
25 kDa
20 kDa
15 kDa
10 kDa
2nd exposure
25 kDa
20 kDa
15 kDa
10 kDa
hSSB2
37 kDa
25 kDa
20 kDa
15 kDa
10 kDa

Supplementary Figure 2

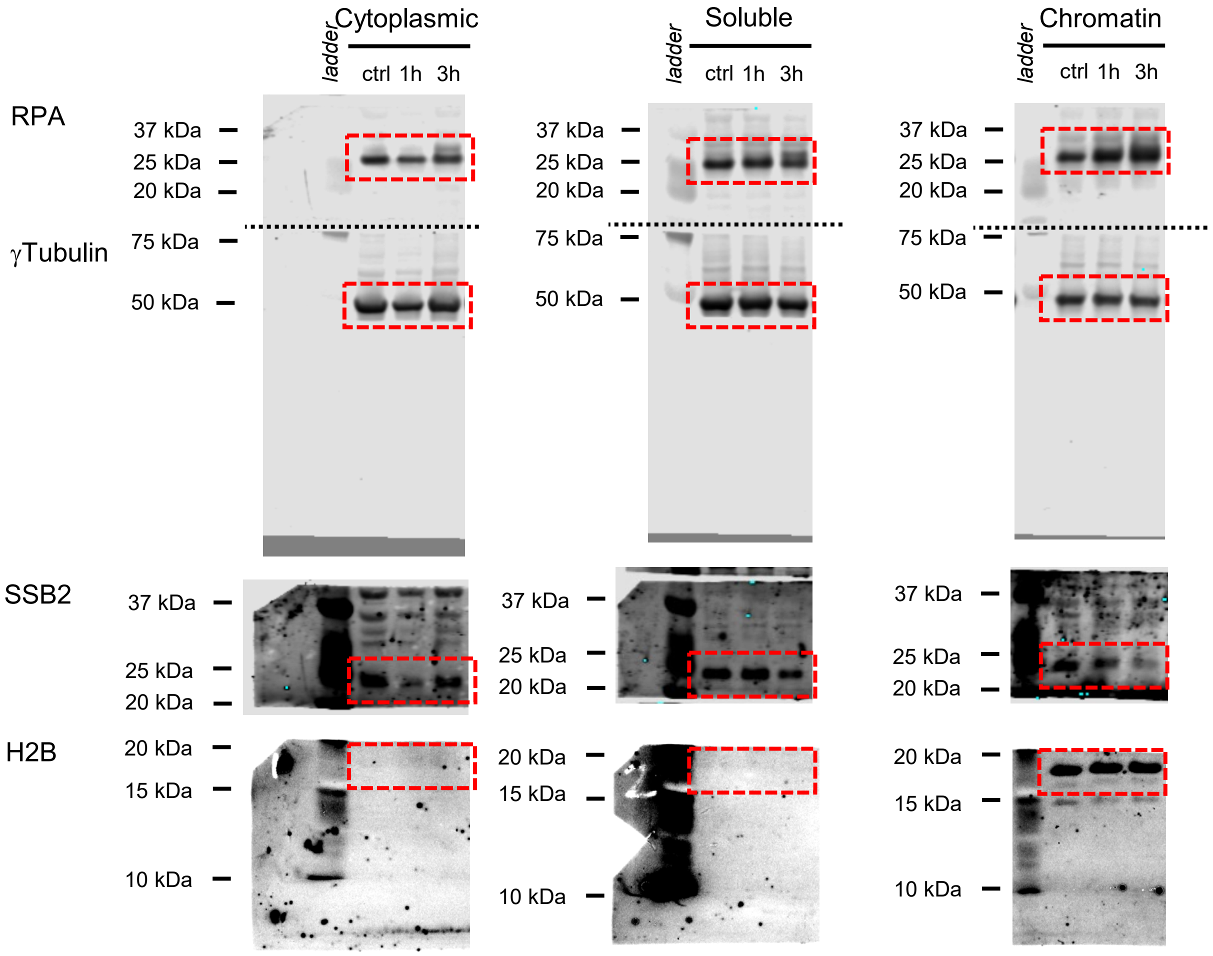

Figure 4b

Top panel

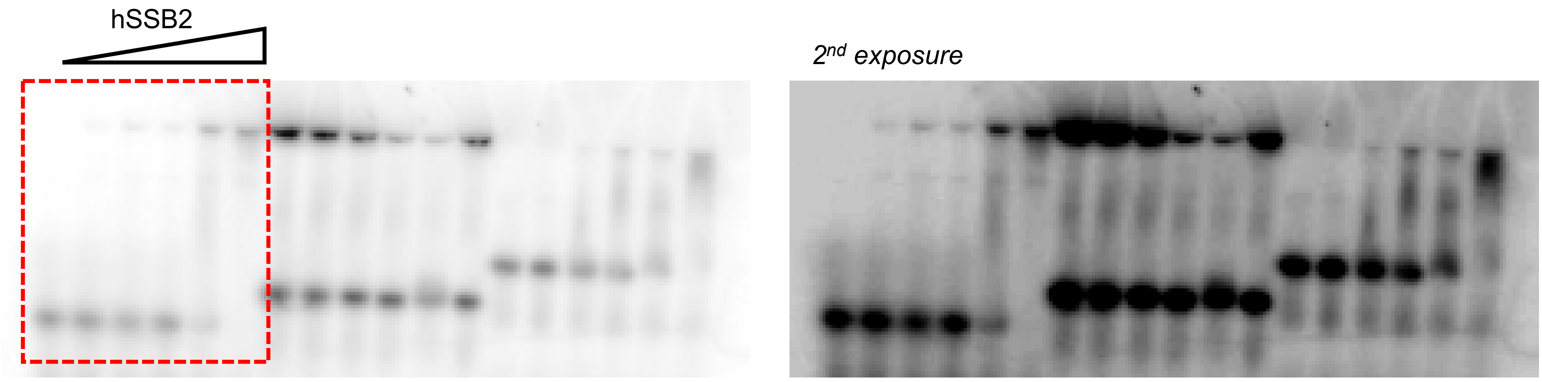

Bottom panel

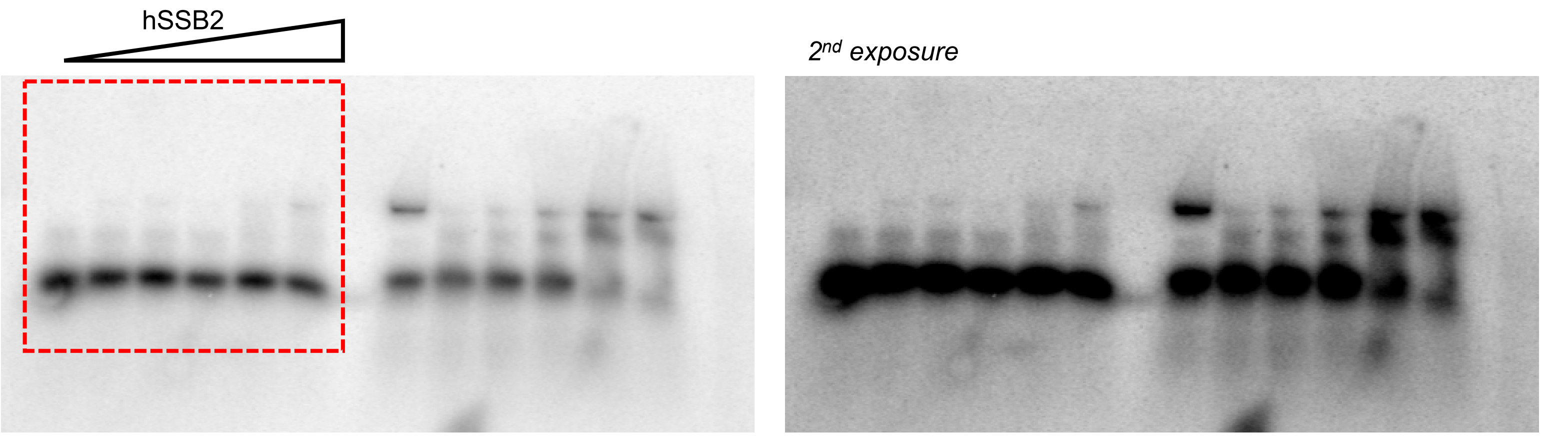

Figure 4c

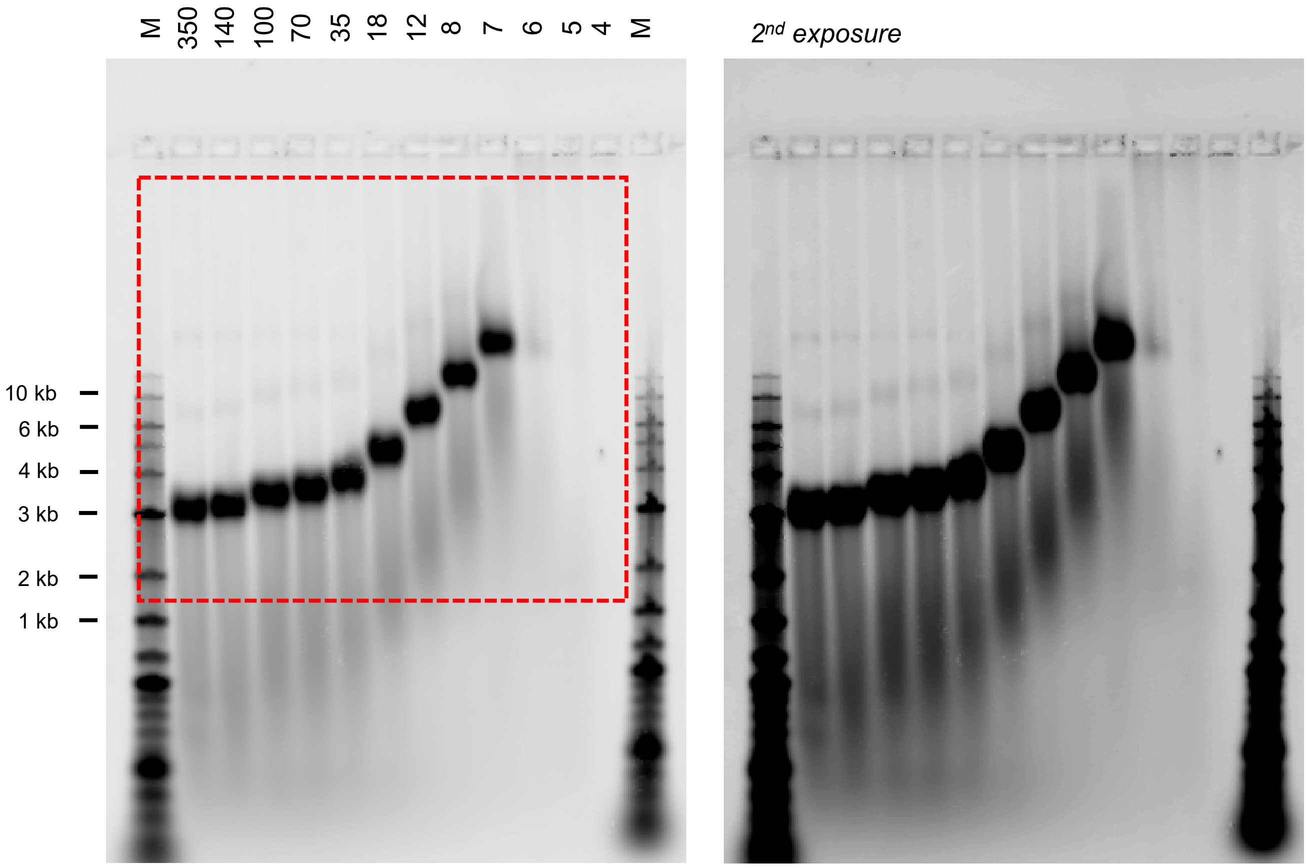

Supplementary Figure 3

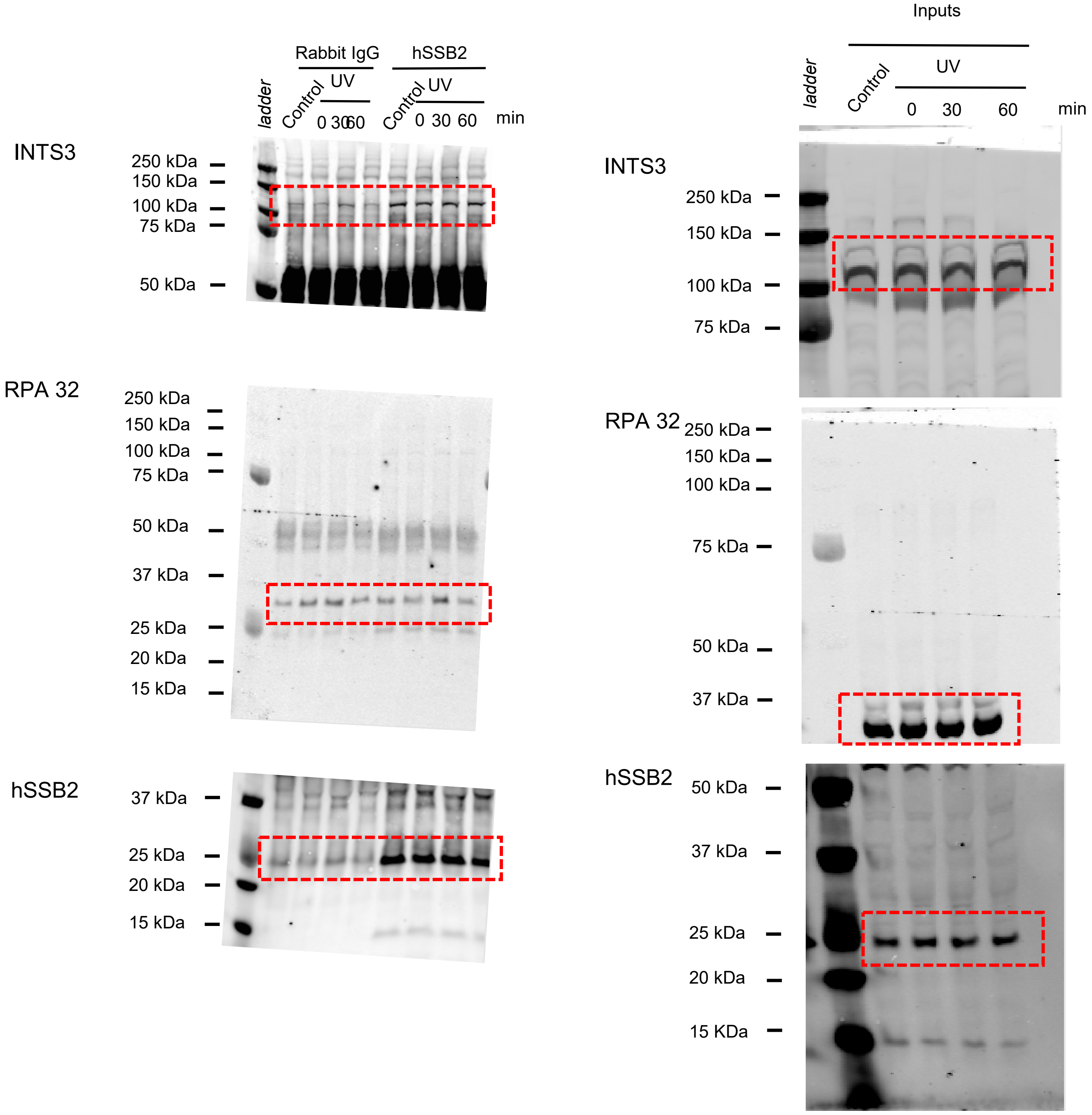

Supplementary Figure 4b

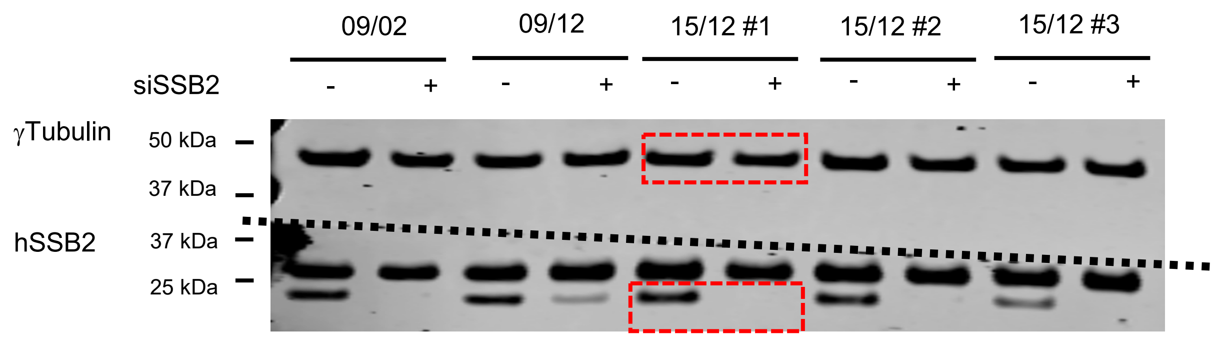

Supplementary Figure 5

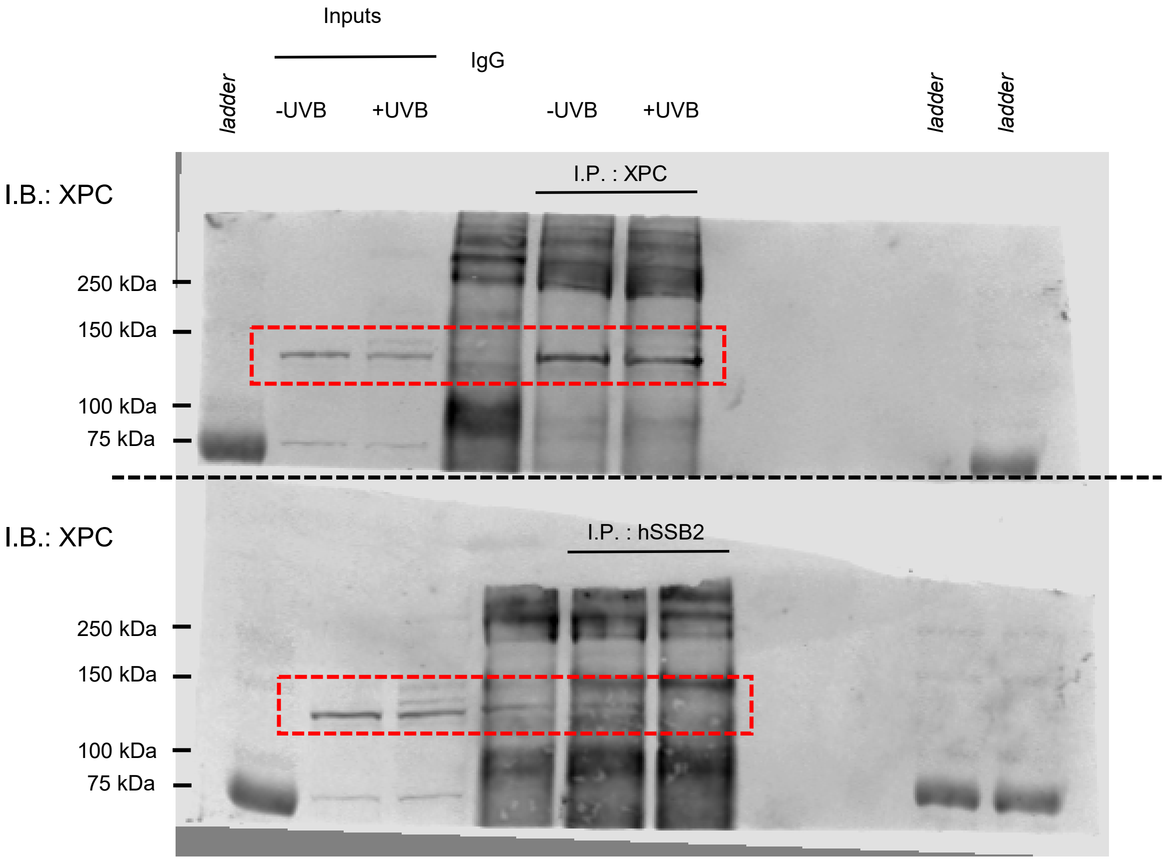

Supplement: Supplementary file 1 — Supplementary Information. [file 41598_2021_99355_MOESM1_ESM.pdf]
